# Compulsive carnival song whistling following cardiac arrest: a case study

**DOI:** 10.1186/1471-244X-12-75

**Published:** 2012-07-03

**Authors:** A Rosaura Polak, Jasper W van der Paardt, Martijn Figee, Nienke Vulink, Pelle de Koning, Miranda Olff, Damiaan Denys

**Affiliations:** 1 Department of Anxiety Disorders, Academic Medical Center (AMC), University of Amsterdam (UvA), Meibergdreef 5, Amsterdam, 1105 AZ, the Netherlands; 2 Emergency psychiatric hospital, Arkin Mental Health Care, 1e Constantijn Huygensstraat, 38, Amsterdam, 1054 BR, the Netherlands; 3 Arq Psychotrauma Expert Group, Nienoord 5, Diemen, 1112 XE, the Netherlands; 4 The Netherlands Institute for Neuroscience (NIN), an institute of the Royal Netherlands Academy of Arts and Sciences, Meibergdreef 47, Amsterdam, 1105 BA, the Netherlands

**Keywords:** OCD, Brain damage, Compulsive, Impulsive, Treatment, SRIs

## Abstract

**Background:**

Compulsivity is the repetitive, irresistible urge to perform a behavior, the experience of loss of voluntary control over this intense urge and the tendency to perform repetitive acts in a habitual or stereotyped manner. Compulsivity is part of obsessive-compulsive disorder (OCD), but may occasionally occur as stand-alone symptom following brain damage induced by cardiac arrest. In this case report, we describe a patient who developed compulsivity following cardiac arrest. We review diagnostic options, underlying mechanisms and possible treatments.

**Case presentation:**

A 65-year-old man presented at our clinic with continuous compulsive whistling following cardiac arrest. Neither obsessive-compulsive symptoms, nor other psychiatric complaints were present prior to the hypoxic incident. An EEG showed diffuse hypofunction, mainly in baso-temporal areas. Treatment with clomipramine resulted in a decrease of whistling.

**Discussion:**

This case report illustrates de novo manifestation of compulsivity following cardiac arrest and subsequent brain damage and gives additional information on diagnostic options, mechanisms and treatment options. Differential diagnosis between stereotypies, punding, or OCD is difficult. Compulsivity following brain damage may benefit from treatment with serotonin reuptake inhibitors. This finding enhances our knowledge of treatments in similar cases.

## Background

Psychiatric symptoms often occur following structural lesions to the brain such as cerebral infarction. Depression [1,2] and generalized anxiety disorder [1,3] are most prevalent, though compulsivity may appear occasionally. Compulsivity consists of the following features: the repetitive, irresistible urge to perform a behavior, the experience of loss of voluntary control over this intense urge and the tendency to perform repetitive acts in a habitual or stereotyped manner [4,5]. Impulsivity is characterized as the tendency to act prematurely without foresight, resulting in behavioral disinhibition. This behavior is sometimes difficult to distinguish from compulsivity due to their phenomenological similarities.

Differentiating between impulsivity and compulsivity and differentiating between disorders that are related to impulsive or compulsive behaviors may help optimize treatment for reducing psychiatric symptoms that may occur following a cerebral infarction. Repetitive behavior can occur in many disorders or conditions, such as frontal syndrome [6], punding [7] or obsessive-compulsive disorder (OCD) [8], but also in autism spectrum disorders and tic disorders [9]. The differentiation between these conditions is not always clear-cut due to phenomenological similarities between impulsivity and compulsivity. For example, where punding is defined as repetitive, meaningless compulsive behavior, this is also characteristic for OCD symptoms. And as frontal syndrome can often occur following infarction, compulsive symptoms may incorrectly be confused with impulsivity related to frontal syndrome.

The purpose of this paper is to provide a detailed case description of repetitive whistling following cardiac arrest and subsequent post-anoxic frontal syndrome. We will discuss differential diagnoses, neuroanatomy and effective treatments.

## Case presentation

In February 1992, Mr. E., at that time 42 years old, was found unconscious in his car. He had suffered myocardial infarction and was reanimated at the emergency room (ER) in Den Bosch, where he was presented with ventricle fibrillation and unresponsive, dilated pupils. He was reanimated successfully, but during rehabilitation Mr. E. was found to be bradyphrenic, disoriented and apathetic. A computerized tomography (CT) scan showed no major lesions, but an EEG showed diffuse hypofunction, especially in the baso-temporal areas. Patient’s short-term memory was severely disturbed and his mood was dysphoric. Neuropsychological examination one year after the cardiac arrest showed less spontaneous speech and disturbed memory encoding and retrieval. However, planning and language were unaffected and the intellectual functioning was rated above average. His close relatives noticed inadequate social behavior and signs of disinhibition, such as sexual references, spitting and continuous whistling.

In the course of a few years, despite a physical and cognitive revalidation program, the cognitive impairments did not improve, and even though most of the disinhibition disappeared, the whistling persisted.

We were approached in May 2008 by the patient’s wife, who got to know our center of expertise through the internet. She was close to desperation from listening to the whistling of the same carnival song for nearly 16 years. It would go on for 5 to 8 hours every day and got worse when the patient was tired. He had been treated with clomipramine 150 mg/day for nearly two months which resulted in a 50% decrease of compulsive whistling (3–4 hours a day), however, with urinary incontinency as an unacceptable side effect. Subsequent treatment with paroxetine 20 mg/day resulted in only a slight relief of the symptoms with similar side effects as with clomipramine.

We visited patient and his wife in their home and were immediately confronted with the clear and perfectly in tune whistling of the same song, almost without interruption. The patient denied anxiety or obsessive thoughts that might instigate his compulsive whistling. He did feel annoyed and anxious if he was asked to stop, on the other hand he told us it would be nice if he would not feel the urge to whistle. Psychiatric evaluation showed a friendly, slightly bradyphrenic man, with severely disturbed cognitive functions such as attention, orientation and short-term memory difficulties. Neuropsychological examination was performed in 1993 (amotivation, slow thinking, memory dysfunction (encoding and retrieval), but above-average intellectual functioning and normal planning, language and calculating) and in 2008 (less spontaneous speech, a disturbed fist palm edge task, go-no go difficulties and disturbed word fluency), mostly showing executive functioning and motivational disturbances. His wife, being the main sufferer from his cognitive impairments, was not only troubled by the whistling but also had to cope with the grief of losing the joyful and sociable husband she married. Before the cardiac arrest she had never noticed any anxious, compulsive or impulsive behaviors. Psychiatric and family history offered no significant details. Aside from mild hypercholesterolemia he had always been physically healthy.

## Discussion

This paper presents a case of a 65-year old patient with compulsive whistling for almost 20 years following a myocardial infarction. The case confronts us with three major questions. Firstly, is the repetitive whistling directly related to a frontal syndrome, punding or secondary to OCD and should it be understood as impulsive or compulsive? As mentioned previously, repetitive behavior can also occur in other disorders such as autism spectrum disorders and tic disorders. However, as the patient never experienced any compulsive, anxious or impulsive behaviors prior in his life, it is very unlikely that the repetitive behaviors are related to a disorder in the autistic spectrum. Nor is the repetitive behavior likely to relate to tic disorders, i.e. Tourette syndrome, with lack of multiple motor tics [9]. Therefore we will only take into account frontal syndrome, punding or OCD. Secondly, which neuroanatomical areas may have caused his compulsive whistling and which brain mechanisms may be related to this repetitive behavior? And finally, what is the optimal treatment to reduce these symptoms?

Firstly, the repetitive whistling may have a merely impulsive nature with disinhibitive behavior. In that perspective, the whistling may be attributed to a frontal syndrome. This syndrome was observed immediately following the cardiac arrest and resulted in a limited period of sexually disinhibited behavior and spitting. The clinical features of the frontal syndrome showed similarities with frontotemporal dementia that is also characterized by disinhibition and actions without foresight of the consequences, such as increased risk-taking behavior [6] and sometimes compulsions with the inability to inhibit repetitive, irresistible urges to perform behavior [10]. Frontal syndrome is associated with structural lesions in the frontal brain areas. Interestingly, simple compulsive behaviors (such as verbal and motor repetitions) seem to correlate with striatal dysfunction, whereas more complex (intentional or time-consuming) behaviors seem to be related to frontal and temporal lobe atrophy, probably reflecting dysfunctional suppression of compulsive thoughts and behaviors [11]. This is in line with the finding that orbitofrontal-basal ganglia dysfunction is associated with OCD [8,12]. In our case, it is unlikely that the whistling is part of a frontal syndrome solely, since it persisted long after the typical disinhibition symptoms spontaneously remitted. The whistling may also be merely impulsive, stereotypic behavior and occur as a stand-alone symptom following brain damage. However, stereotypies are not accompanied with premonitory urges, internal desire to perform and relief from these urges [13]. As our patient clearly feels anxious when stopped and feels the urge to perform, this is not likely to be stereotypic behavior alone.

Another possibility is that the repetitive whistling may be understood in the context of compulsivity and may be associated with punding. Punding is characterized by purposeless and repetitive behavior, such as collecting or arranging things [7,14] often related to the patients’ personal hobbies or occupation and attributed to alterations of the brain’s reward and motor systems in both the ventral and the dorsal striatum [15]. Several aspects of the compulsive whistling show similarities with punding: its repetitive and somewhat comforting character, the irritability and dysphoria when interruption was attempted, and the fact that our patient used to like singing this song in his role as head of a carnival association. Mostly, punding is associated with dopamine dysregulation syndrome (DDS) and occurs as a consequence of therapy with dopamine agonists. More specifically, the punding phenomenon is directly related to a dopamine dysregulation with a clear link to dopamine agonists and L-dopa addictive behavior. A relation with drugs that interact with serotonin receptors, such as quetiapine, have occasionally been found to induce punding [15]. However, sometimes punding is not directly related to chemical induction or hypersensitivity, but more closely related to structural abnormalities. One particular case of compulsive singing as part of dopamine dysregulation syndrome in Parkinson’s disease shows a striking clinical resemblance with our case [15]. Nguyen et al. [16] reported a case in which punding was observed after infarction.

One may hypothesize, thirdly, that the repetitive whistling has a compulsive character and may be originated in the development of OCD. Specific lesions are known to be involved in OCD, such as frontal regions and the basal ganglia. OCD has been related to a decreased volume in these areas, to abnormal neuronal activity in the orbitofrontal cortex, the anterior cingulate, the dorsolateral prefrontal cortex, the caudate nucleus and the thalamus [8]. Also dysfunctional neurotransmitter pathways [17] are suggested to be involved, specifically serotonergic, dopaminergic and glutaminergic pathways with serotonergic reuptake inhibitors (SRIs) as first-line treatment in OCD and tricyclic antidepressants (TCAs), in particular clomipramine, proved to be effective. Typically, obsessive-compulsive symptoms start gradually, early in life and persist. They do not frequently occur following an infarct. Some case studies, however, report a late and sudden onset of OCD symptoms following an organic etiology in which orbitofrontal areas and/or basal ganglia are harmed [18-20]. Like idiopathic OCD, acquired OCD after brain injuries often responds well to SRI treatment [19,21], which was true for our case. Some other studies however, show less successful treatment outcome [22,23]. Our patient did not suffer from typical anxiety symptoms of OCD, however, anxiety is often lacking in other cases of acquired OCD after brain injuries and may be more of a secondary phenomena in OCD. Finally, this particular case does not present with obsessions that are usually preceding compulsive behaviors in OCD.

This case of repetitive whistling shows similarities with a frontal syndrome, punding and OCD. However, it does not fit all features of these diagnoses and may be present as an independent symptom. Furthermore, one may conclude that the whistling with its repetitions is primarily compulsive rather than impulsive or disinhibitive, as the patient had a constant urge to whistle and felt anxiety when asked to stop rather than acting without foresight. The fact that anxiety was felt is in line with compulsivity rather than impulsivity, assuming that compulsive behaviors are performed to prevent perceived negative consequences to happen [4]. In impulsivity, the tendency to act prematurely without foresight would be more prominent and anxious feelings are not necessarily present in impulsive behaviors with diminished regard to negative consequences. Interestingly, not only OCD but also punding [15] and features of frontal syndrome, i.e. frontotemporal dementia [10] may show compulsive symptoms that are attributed to disturbances in the cortico-striatal pathway.

The current case shows that clomipramine decreased the repetitive whistling. Interestingly, SRIs may be effective in alleviating symptoms of compulsivity in idiopathic [17] and acquired [19,21] OCD, frontal syndrome [24] and punding [15]. This suggests that compulsive symptoms may be effectively treated with SRIs or TCAs, even when compulsivity occurs as a stand-alone symptom.

In conclusion, this case shows that the whistling can be explained in the context of compulsivity with its repetitive character. It illustrates that the compulsive behavior can be present as an independent symptom of cortico-striatal dysfunction, and may not always belong to frontal syndrome, punding or OCD. Finally, this case illustrates that pharmacological treatment with clomipramine is effective and suggests that similar cases of compulsivity may benefit from this treatment.

## Consent

Written informed consent was obtained from the patient for publication of this case report.

## Abbreviations

OCD = Obsessive-compulsive disorder; EEG = Electroencephalogram; ER = Emergency room; CT = Computerized tomography; DDS = Dopamine dysregulation syndrome; SRIs = Serotonin reuptake inhibitors; TCAs = Tricyclic antidepressants.

## Competing interests

The authors declare that they have no competing interests.

## Authors’ contributions

ARP and JWP reviewed the literature and wrote the report. JWP, MF and DD followed up the patient during treatment. NV, MF, PK and MO took part in the scientific discussion and contributed to writing the case presentation. DD conceptualized and supervised the procedure. All authors read and approved the final manuscript.

## Pre-publication history

The pre-publication history for this paper can be accessed here:

http://www.biomedcentral.com/1471-244X/12/75/prepub
